# High-Q Wafer Level Package Based on Modified Tri-Layer Anodic Bonding and High Performance Getter and Its Evaluation for Micro Resonant Pressure Sensor

**DOI:** 10.3390/s17030599

**Published:** 2017-03-16

**Authors:** Liying Wang, Xiaohui Du, Lingyun Wang, Zhanhao Xu, Chenying Zhang, Dandan Gu

**Affiliations:** 1 Department of Mechanical and Electrical Engineering, Xiamen University, Xiamen 361005, China; wangliying@stu.xmu.edu.cn (L.W.); 19720142203279@xmu.edu.cn (Z.X.); 19720142203256@xmu.edu.cn (C.Z); 2 Instrumentation Technology & Economy Institute, Beijing 100055, China; dxh@instrnet.com; 3 Pen-Tung Sah Institute of Micro-Nano Science and Technology, Xiamen University, Xiamen 361005, China

**Keywords:** micro resonant pressure sensor, high quality factor, cross-layer anodic bonding, Ti getter, wafer level package

## Abstract

In order to achieve and maintain a high quality factor (high-Q) for the micro resonant pressure sensor, this paper presents a new wafer level package by adopting cross-layer anodic bonding technique of the glass/silicon/silica (GSS) stackable structure and integrated Ti getter. A double-layer structure similar to a silicon-on-insulator (SOI) wafer is formed after the resonant layer and the pressure-sensitive layer are bonded by silicon direct bonding (SDB). In order to form good bonding quality between the pressure-sensitive layer and the glass cap layer, the cross-layer anodic bonding technique is proposed for vacuum package by sputtering Aluminum (Al) on the combination wafer of the pressure-sensitive layer and the resonant layer to achieve electrical interconnection. The model and the bonding effect of this technique are discussed. In addition, in order to enhance the performance of titanium (Ti) getter, the prepared and activation parameters of Ti getter under different sputtering conditions are optimized and discussed. Based on the optimized results, the Ti getter (thickness of 300 nm to 500 nm) is also deposited on the inside of the glass groove by magnetron sputtering to maintain stable quality factor (Q). The Q test of the built testing system shows that the number of resonators with a Q value of more than 10,000 accounts for more than 73% of the total. With an interval of 1.5 years, the Q value of the samples remains almost constant. It proves the proposed cross-layer anodic bonding and getter technique can realize high-Q resonant structure for long-term stable operation.

## 1. Introduction

The resonant pressure sensor [1], which is one of the best pressure sensors with the highest accuracy and long-term stability, plays a very important role in the field of aerospace, weather detection, industrial process control, and other precision measurement [2,3]. It can indirectly measure pressure by measuring the change in resonant frequency of the resonant beam, since the deformation of the sensitive membrane caused by the change in pressure changing the resonant frequency of the double-ended clamped resonant beam. The high-performance obtaining of the resonant pressure sensor is closely related to the degree of vacuum package, since the resonant beam is subjected to air damping to dissipate kinetic energy that leads to Q value reduction of the device and affects the performance of the device in turn. In other words, high vacuum package degree is an important guarantee for the normal vibration of the resonant beam and the high-Q value of the sensor. Two important indicators to evaluate the quality of the vacuum package are the vacuum degree and its long-term stability. There are many ways to achieve high vacuum package degree and maintain vacuum stability of the device, such as choosing dense packaging materials, improving package structure [4], fully baking before bonding, adopting a dry process in the process steps as much as possible, better packaging with less deflation [5], and introducing the getter [6]. The above mentioned method shows that there are two ways to achieve high vacuum package: one is to reduce the vacuum degree of the air source initiatively; another is the passive absorption gas to improve the vacuum degree.

Wafer-level package is an effective way to achieve a low vacuum degree initiatively with minimal packaging costs and high reliability [7,8]. There are already several well-established wafer-level package techniques for resonant pressure sensors, such as silicon direct bonding (SDB) [9], glass frit bonding [10], and anodic bonding [11]. Anodic bonding is widely used in micro-electromechanical systems (MEMS) because of its high bonding stability and simple bonding equipment. However, the resonant pressure sensor fabricated by bulk silicon process usually is a structure with three or more layers. Although the single-step anodic bonding [12] can achieve triple layers (tri-layer) anodic bonding, it can affect the bonding quality due to the follow-up bonding being unable to achieve the same quality as the preceding bonding. Apart from the single step anodic bonding, another two methods have been proposed for the tri-layer structure: (1) bonding by three electrodes [13] and (2) bonding by two-electrodes with one pole reversal [14]. Both techniques improve the efficiency of tri-layer bonding, but are inappropriate for acquiring a good performance for the package because of the stacked wafers in advance and bonding defects after reversal. Additionally, through-hole has been demonstrated to be an effective method to achieve the same bonding quality as the Silicon/Glass (GS) bonding [15]. However, it requires one more through hole for one more layer, and the interface of the bonding layer is exposed by through holes that make the method interfere with other MEMS processes. In general, in order to achieve a higher vacuum package degree of the resonant pressure sensor by bulk process, the tri-layer anodic package with a simple process and high reliability still needs to be studied and solved.

Adding the getter is a way of increasing the vacuum degree by the passive absorption of the gas. Classically, M. Hasegawa [16] applied PageWafer getter to the atomic clock, mainly to analyze the residual gas composition in the chamber after package. The result is given in Table 1. The related indicators such as mass spectrometer and atomic clock relaxation time adopted in the paper proved anodic bonding to be a typical packaging method. Anodic bonding is a class of gas-releasing process, and the introduction of getter can effectively reduce the amount of gas in the chamber in order to obtain and maintain high vacuum. However, the volume of the packaging cavity is generally very small for one typical micro-electromechanical system (MEMS) sensor which makes it very difficult to put more getters in the cavity. Therefore, the means used to optimize the performance of the getter becomes ever more important. The research associated with the performance improvement on getter, active condition optimizing, and getter integration in the packaging processes for resonant pressure sensors is rarely considered in previous literature.

In this paper, one modified tri-layer anodic bonding method is presented for high-vacuum package and long-term stability of micro resonant pressure sensor. Owing to the problems of poor bonding quality and complicated process caused by single-step tri-layer anodic bonding, the cross-layer anodic bonding technique was adopted to achieve high vacuum package degree by sputtering aluminum (Al) on the combination wafer of the pressure-sensitive layer and the resonance layer after bonding them with SDB to achieve electrical interconnection. The model of the cross layer anodic bonding was established and the bonding result was tested to verify that the provided bonding method can realize electrical interconnection with high bonding quality. On the other hand, the titanium (Ti) getter was also introduced into the vacuum cap layer to maintain the vacuum package degree after optimizing its preparation process. Finally, Q value test system was built, and Q value and its long-term stability were also tested in this paper.

## 2. Brief Introduction of the Resonant Pressure Sensor

**Figure 1:**
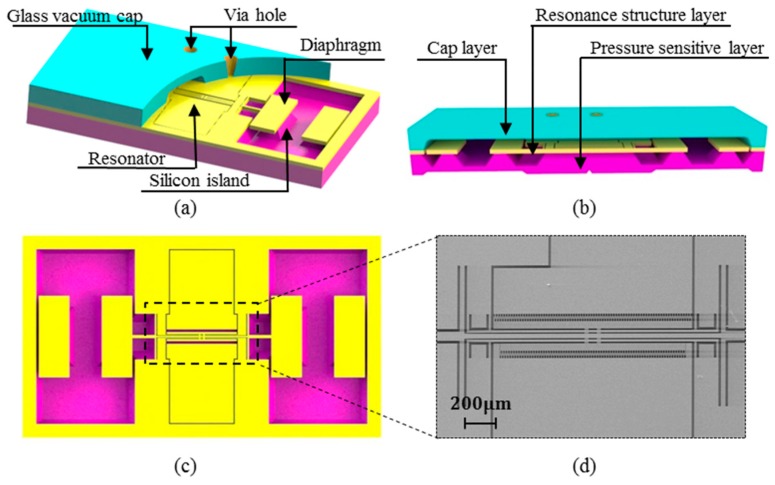
Schematic of the designed resonant pressure sensor without electrode wire layer. The blue, yellow, and pink layers represents the glass vacuum cap layer, the resonance structure layer, and the pressure sensitive layer, respectively. (**a**) Sectional view of the main surface of the resonant pressure sensor; (**b**) Sectional view of the resonant pressure sensor; (**c**) The resonance structure layer with each diaphragm attached to one end of the resonator by a silicon island; (**d**) The stable resonator for frequency output.

The structure diagram of the designed double membrane micro silicon resonant pressure sensor is shown in Figure 1a. The sensor is divided into four layers: the top electrode wire layer with driving and detection electrodes of the resonance structure and grounding electrode of the movable structure, the vacuum gap layer with a via hole for the electric connection, the resonance structure layer, and the bottom pressure sensitive layer with dual symmetrical diaphragm (Figure 1b shows without electrode wire layer). Each diaphragm is attached to one end of the resonator by a silicon island. The designed resonant pressure sensor, based on electrostatic-driven and capacitive detection, recognizes the change of external pressure with the change of output frequency, and its pressure sensitive function is achieved as follows: First, the resonance structure resonates under alternating current (AC) driving signal, and then pressure sensitive film is deformed when the external pressure increases, leading to more pressure on the resonance beam. Finally, the resonance frequency of the resonance beam is reduced with a reduction in stiffness. It has two main functionality parts: (1) the diaphragms (Figure 1c) and the silicon island for force transmission and (2) the stable resonator (Figure 1d) for frequency output. The silicon island can increase the sensing sensitivity without large the out-of-plane displacement of the vibrating beams compared to the sensor fabricated with silicon-on-insulator (SOI) [17].

The resonant sensor was micro fabricated with MEMS bulk technology on a 4-inch wafer level. Silicon anisotropic wet etching with tetramethyl ammonium hydroxide (TMAH), and the SiO_2_ mask was adopted to handle the pressure-sensing diaphragm and the transmission silicon island of the Si pressure sensitive layer with a back protection fixture. The Si resonant layer, with a thickness of 80 μm, was processed by a photoresist mask and deep reactive ion etching (DRIE) after lapping and chemical mechanical polishing (CMP) to fabricate the resonator and the different beams. Pyrex 7740 glass wafer was chosen as the material of the vacuum cap layer. The via hole on it was made by laser and the driven and sensing electrodes were attached to the top surface of the glass cap by Al sputtering. The fabrication error of the resonant structure and diaphragm is controlled within ±1 μm and ±2 μm, respectively, and the percent of the pass of Al electrodes is approaching 100%.

## 3. Cross-Layer Anodic Bonding Technology

### 3.1. Process Analysis and Model of the Cross-Layer Anodic Bonding

**Figure 2:**
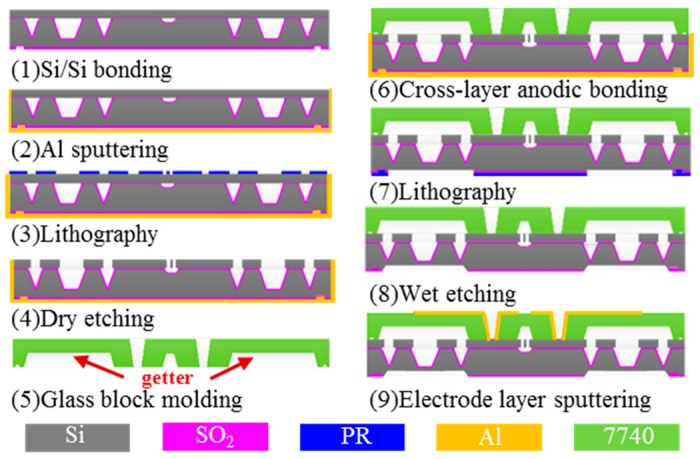
Schematic of the proposed tri-layer wafer level package with cross-layer anodic bonding and getter integrated.

Electrical connection and effective ohm contact are the basis for realizing the electromechanical coupling of the sensitive chip. Due to the problems of poor bonding quality and complicated process brought by tri-layer anodic bonding of single step, this paper presents one modified tri-layer anodic bonding process to achieve and maintain high-Q of the micro resonant pressure sensor shown in Figure 2. Here, the preparation process of the glass block, the resonance structure layer, and the pressure-sensing layer are not listed. First, SDB was applied to attach the pressure-sensing layer and the Si wafer after the pressure-sensing layer formed with silicon anisotropic wet etching under the SiO_2_ mask and a back protection. The insulation SiO_2_ layer between the two layers was 0.3 μm. Then the resonant layer was processed by a photoresist mask and DIRE after CMP. After supttering Al at a vacuum of 10^−3^ Pa on the combination wafer of the pressure-sensing layer and the resonant layer, the combination wafer was subjected to anodic bonding with the formed glass cap in accordance with the conditions of the anodic bonding. It is worth noting that in order to prevent the oxidation of Al in the bonding process, the bonding machine was pumped into a vacuum of 10^−3^ Pa before heating, compression and alignment, and the bonding machine was also filled with inert N_2_ gas during the cooling period. Then the pressure-sensing diaphragm was formed by wet etching after an SiO_2_ corroded by buffered oxide etch (BOE) solution. Finally, the tri-layer wafer level package was finished after the top electrode wire layer was formed by sputtering Al with a hard mask. The cross-layer anodic package of the patterned glass block and the pressure-sensing layer is the main step necessary to form a vacuum atmosphere in the resonant structure.

**Figure 3:**
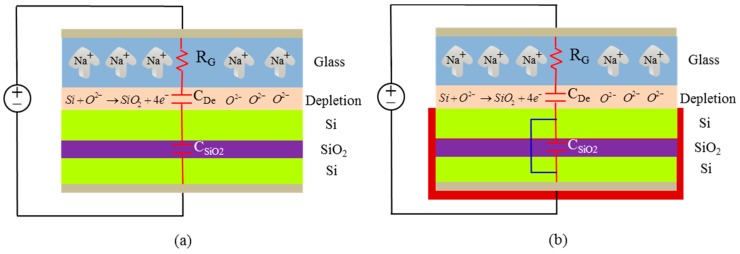
(**a**) The configuration and the associated electrical model of the single step GSS anodic bonding; (**b**) The configuration and the associated electrical model of the cross-layer anodic bonding. The Si/Si composite wafer is short out by a sputtering metal interconnection.

In order to verify the feasibility of the proposed cross-layer anodic bonding, the configuration and the associated electrical model of the glass/silicon/silica (GSS) anodic bonding with single step [18] and cross-layer step were established, as shown in Figure 3a,b, respectively. During the tri-layer anodic bonding with single step, the glass wafer was a feeble conductor that was equivalent to a resistor, R_G_. The depletion layer at the junction of glass and silicon were equivalent to a capacitor, C_De_. The thermal oxide layer was almost completely insulator since it did not have any special doping, such as glass wafer doped sodium (Na) atoms, to provide sufficient conductivity. Therefore, it could not be modeled as a weak conductor like the glass wafer. Here, the silica layer (SiO_2_) was modeled as a fixed capacitor, C_SiO2_. In the beginning, the bonding voltage directly applied on the depletion layer, and the SiO_2_ layer acted as a short circuit. With the passage of time, the C_SiO2_ was charged by the bonding current and dielectric break down did not take place in the SiO_2_ layer since the bonding potential across C_De_ droped continuously to a value as the voltage was shared between C_SiO2_ and C_De_. The bonding potential of the stacked GSS bonding was higher compared to the standard glass/Silicon (GS) anodic bonding since the applied voltage was divided between the SiO_2_ and depletion capacitances. In the other words, it was hard to obtain the quality as the GS anodic bonding.

However, if the Si/Si (SS) composite wafer is short out by a sputtering metal interconnection, the effect of the partial voltage of the insulating SiO_2_ layer can be eliminated. The existed impedances C_SiO2_ in bonded wafers is bypassed by the metal, thus, “shorting out” bonding can efficiently resolve the “voltage division” dilemma emerging in single-step anodic bonding. That is the “shorting out” principle of cross-layer anodic bonding. Possibly, the cross-layer anodic bonding can successfully achieve the similar bonding performance as GS anodic bonding.

### 3.2. Discussion of the Bonding Effect

**Figure 4:**
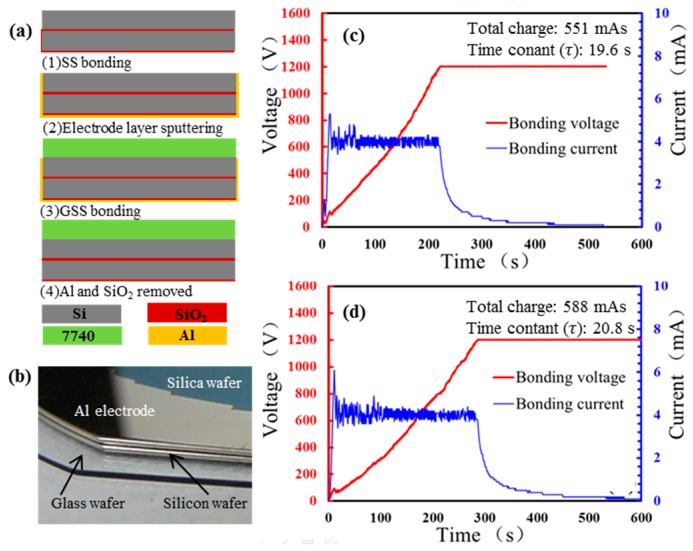
An experimental bonding process was conducted to verify the correctness and feasibility of the cross-layer anodic bonding. (**a**) The Schematic of the test process; (**b**) The bonding result of the cross-layer anodic bonding; (**c**) The anodic bonding electrical characters of the GSS cross-layer anodic bonding; (**d**) The anodic bonding electrical characters of the GS anodic bonding.

In order to verify the possibility as mentioned above, as well as the correctness and feasibility of the cross-layer anodic bonding model, an experimental bonding process, as showed in Figure 4a, was developed. The key point of the process was the preparation of the electrode and it was achieved by magnetron sputtering which is relatively simple. MYCRO’s AWB04 chip bonding machine (Applied Microengineering Ltd., Oxfordshire, UK) was employed in SDB and anodic bonding. The JC500-3/D magnetron sputtering machine produced by Chengdu vacuum machine factory (Chengdu, China) was supported in magnetron sputtering. The thickness of the silicon and glass used in the experiment were respectively 500 µm and 400 µm. The electrode was attached on one side of the combination SS wafer. Due to the disordered nature of the magneto-sputtering, the lateral position of the intermediate Si layer was also sputtered with Al, thereby achieving electrical interconnection between the bilayers of silicon, i.e., the previously mentioned “short-circuit” connection. Then the tri-layer anodic bonding was carried out and the temperature, voltage, vacuum degree and mechanical stress were 400 °C, 1000 V, 10^−3^ Pa, and 200 N respectively. Finally, Al was completely washed off by the cleaning of the RCA-3 without damaging the structure.

The bonding result is presented in Figure 4b. No defect was found and the bonded area of wafer was almost 100%. The huge grey area of bonding interface proves the feasibility of the designed GSS cross-layer bonding method. The electrical character of the GSS cross-layer bonding is illustrated in the Figure 4c. In order to verify the “short circuit” performance of the GSS cross-layer anodic bonding, a single step anodic bonding of GS was carried out under the same experimental parameters with the preceding GSS bonding and its electrical characters as shown in Figure 4d. According to the proposed electrical model of the single-step GSS anodic bonding, the current of each step bonding should obey exponential decay, which reflects the capacitor and resistor property of the system. The time constant (τ) in GSS cross-layer bonding has the similar τ with the traditional GS bonding, indicating that they have similar RC circuit, and the sputtered metal achieves the effect of “short circuit” mentioned. On the other hand, total charge responds to the bonding voltage and temperature directly [19], and both parameters impose a positive effect on the magnitude of the total charge. The total charge of one single-step GSS bonding should be less than that of the GS bonding under same bonding condition according to the analysis of the preamble voltage division prediction. The approximate time constant and the total charge of the GS bonding and the GSS cross-layer bonding affirm that the cross-layer bonding has a similar electrical processes by the GS bonding, which verified the correctness of the cross-layer anodic bonding model. The stacked wafer with cross-layer anodic bonding was diced into pieces of 8 mm × 8 mm for tensile strength testing, and it is obtained more than 25 MPa: there was no destruction at the bonding wafer when the epoxy broke. At the same time, a tensile strength test was also made on the tri-layers wafers by a single step bonding process, and the tensile strength was lower than 9 MPa. Strength testing comparing single-step bonding and cross-layer bonding showed the strength of the cross-layer anodic bonding provided sufficient bonding strength for the designed micro resonant pressure sensor.

**Figure 5:**
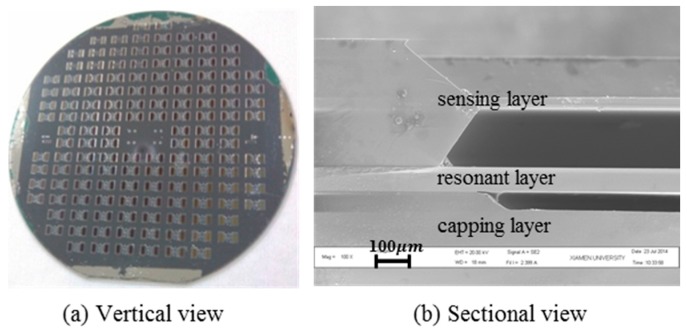
The sensor is after tri-layer anodic bonding by cross-layer. (**a**) The vertical view of the sensor; (**b**) The section view of the sensor.

The vacuum package was completed according to the package process as showed in Figure 2. Before step (6) in Figure 1, the wafers should be baked at 450 °C under vacuum for 1 h to remove the gas adsorbed on the material surface. Then, Si/Si wafer and the glass cap were anodic bonded according to pre-study. The bonding result is given in Figure 5. The whole devices on the 4-inch wafer achieved 100% hermetic package as illustrated in Figure 5a. Taking random optical checks of all devices, the resonant structures were located in the etched groove accurately. The sectional image of the sensor taken by scanning electron microscope (SEM) also showed the shape of the sensor structure guaranteed by tri-layer stackable process as Figure 5b shown.

## 4. Ti-Based Getter Performance Optimization and Integration

### 4.1. Fabrication of Ti-Based Getter and Its Performance Comparison

The high-Q vibration of the resonator cannot be assured by the cross-layer anodic bonding only. The bonded wafer was taken out after natural cooling of about 1 h from 400 °C to 100 °C. During this period, the glass material released a lot of gas because of high temperature baking, resulting in vacuum chamber pressure much higher than 10 Pa. The Q then dropped to thousand. At the same time, the glass material continued to vent, resulting in the temperature immune deterioration of the vacuum microcavity. Therefore, the problem of how to maintain the vacuum degree and avoid the effect of deflation from the internal material of the device on the vacuum inside the device must be solved. The most effective measure to improve and maintain the vacuum degree of the microcavity was to add getter [20] with high inspiratory characteristics.

**Figure 6:**
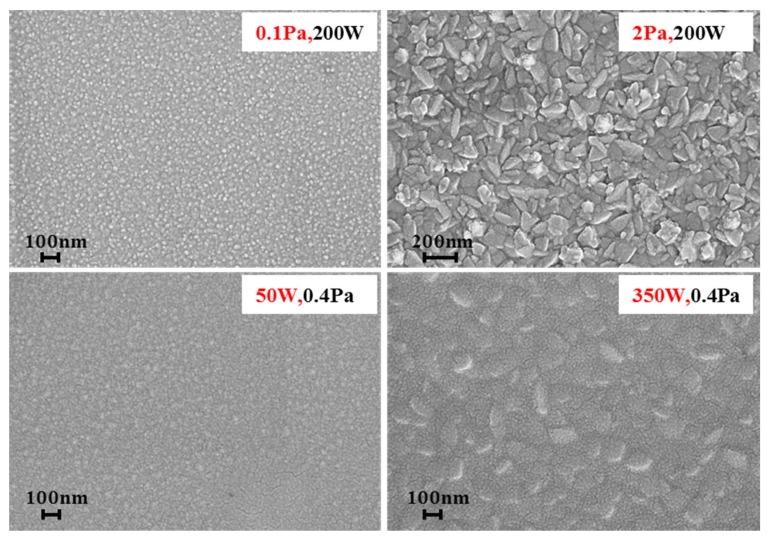
SEM characterization of the surface morphology of the films under different sputtering parameters (with the same multiples of 50 K).

Titanium (Ti) is a getter material that is taken by most of the vacuum package MEMS devices at present [21] because its preparation process is relatively simple and it is more compatible with the entire MEMS preparation process. In this paper, Ti was used as getter material and a getter film was deposited on the inside groove of the glass cap layer by magnetron sputtering. In order to ensure the integration of efficient getter material in a limited groove, the preparation parameters of the getter were investigated to obtain a highly specific surface area before Ti integrated in the sensor. The specific surface area of the Ti film was measured by TriStar II 3020 produced by Micromeritics Company (Norcross, GA, USA). The change of sputtering parameters is mainly aimed at the pressure and sputtering power. The experimental parameters as showed in Table 2 were worked out to explore the influence of different sputtering power and pressure on the morphology of the films. The Ti films sputtered, according to the parameters in Table 2, were characterized by scanning electron microscope (SEM) and the results are shown in Figure 6. It is clear that the increased pressure and power made the film surface roughness and particle size increase, which also suggests improving the surface roughness and the specific surface area to increase the pressure or improve the sputtering power.

**Figure 7:**
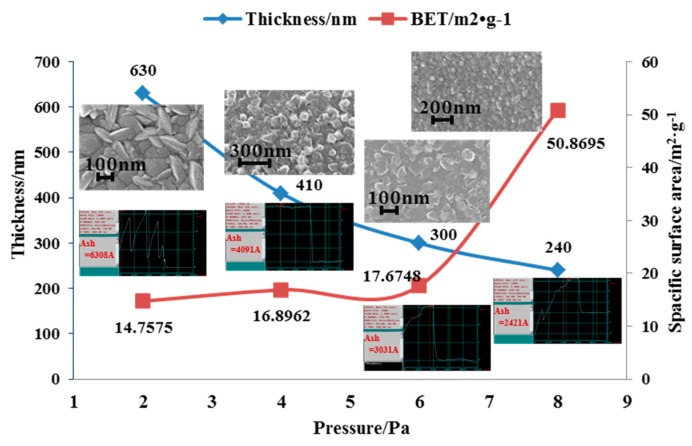
The effects of different sputtering parameters on the specific surface area and thickness of the films.

However, the magnetron sputtering machine used in the experiment had a maximum sputtering power of 350 W, so the specific surface area was supposed to be characterized by changing the pressure. The effects of different pressure on the specific surface area and thickness of the films were investigated under the same sputtering time of 10 min and sputtering power of 350 W. As shown in Figure 7, it can be seen that the specific surface area of the Ti film increased with increasing argon (Ar) pressure while maintaining the sputtering power constant of 350 W, and the magnitude of the increment was maximum at 8 Pa. At low pressure, such as 2 Pa, the Ti film was a columnar crystal with a larger size, and at higher pressure, such as 4 Pa, 6 Pa and 8 Pa, the Ti film formed a more uniform crystal structure. This is mainly due to different air pressure, resulting in altered surface properties of Ti and different morphology. In addition, as the gas pressure increased, the Ti film thickness decreased gradually. This was mainly because the gas molecules mean free path and pressure has the following relationship [22]:

(1) $$\lambda =\frac{kT}{\sqrt{2}\pi d^{2}P}$$

where λ is the mean free path of the gas molecule, k is Boltzmann’s constant, T is the temperature of the gas, d is the diameter of the gas molecule, and P is the pressure. In the case of temperature and gas molecular diameter at the same time, when the chamber pressure increases, the mean free path of a particle decrease and the number of gas molecules increase, resulting in the loss of more energy of the Ti atoms in the deposition process when frequently collide with gas ion. On the other hand, the increase collision of Ti atom and gas ion probability possibly increased the scattering degree of Ti [22]. These two factors reduce the probability of Ti atoms reaching the substrate, thus decreasing the deposition rate of Ti thin films.

### 4.2. Active Condition and Packaging Integration of the Getter

**Figure 8:**
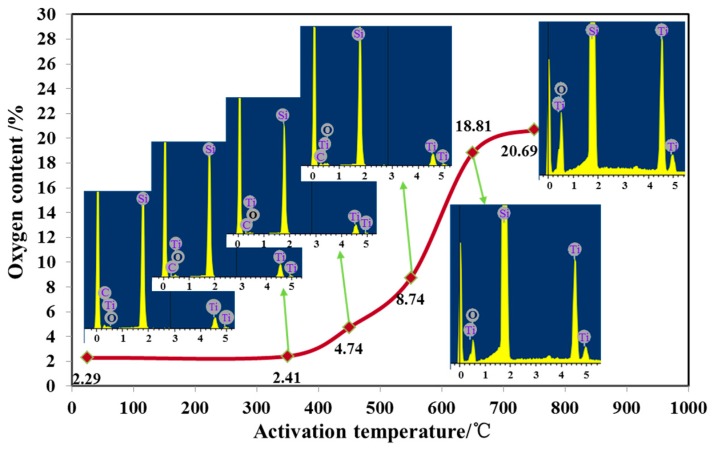
The effect of different activation temperatures on the oxygen content of the films.

**Figure 9:**
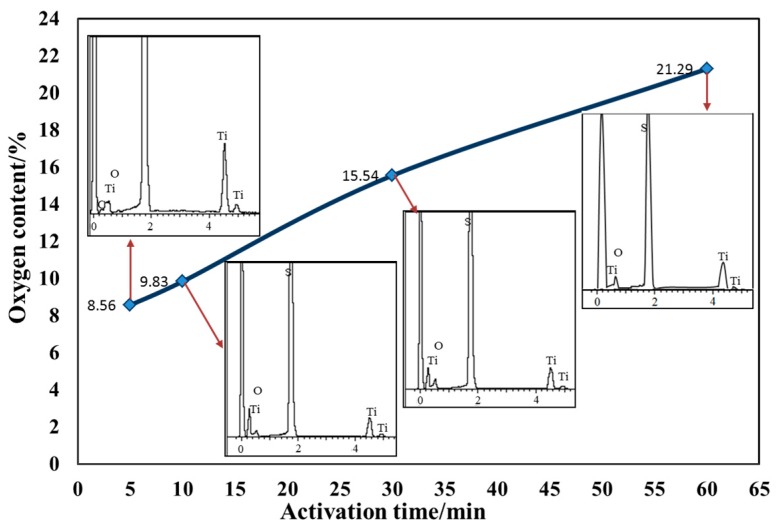
The effect of different activation time on the oxygen content of the films.

The surface of Ti getter can easily form a passivation layer in the air, so the passivation layer needed to be removed to expose its active surface, i.e., the need to activate the getter. The activation and inspiration effects of getter was closely related to the activation temperature and heating time. The getter was activated at different temperatures (350 °C, 450 °C, 550 °C, 650 °C, 750 °C ) at a fixed holding time (30 min) in the vacuum, and the oxygen content of the getter put in the atmospheric environment for 10 h after activation was measured by an Energy Dispersive X-ray Detector (EDX) test. As showed in Figure 8, it can be clearly seen that the oxygen content increased with the increase of the activation temperature, and the suction effect was better. At about 650 °C, the Ti film was completely activated. At this time, the passivation layer was totally destroyed and the inspiration rate was the highest and the effect was the best. Above 650 °C, increasing the activation temperature will not improve the inspiratory effect. The activation temperature was fixed at 600 °C and the activation time was changed to 5 min, 10 min, 30 min, and 60 min, and the heating rate was 10 °C/min in order to study the effect of different activation time on Ti film. In the same way, the oxygen content of the EDX spectrum was utilized to characterize the inspiratory effect. It can be seen from Figure 9 that the oxygen content was in a linear upward trend with the activation time. The optimal activation time was 60 min or more, with the increase of activation time owing to the compactness of the oxide layer on the surface of the pure Ti film.

**Figure 10:**
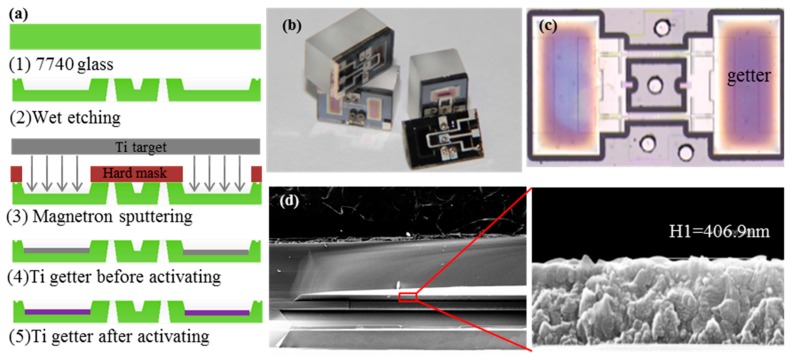
The Ti getter was prepared under optimum conditions. (**a**) The integrating of Ti getter on the inside of the patterned glass cap; (**b**) The wafer-level packaging of the sensor; (**c**) The getter was fully activated after heated for 6 h at 600 °C; (**d**) Enlarged view of the prepared getter film at a magnification of 50 K by SEM.

The optimal process parameters of sputtering Ti were obtained by parameterizing the preparation process and activating process of Ti getter material is 8 Pa, 350 W, and the best activation condition of getter is 600 °C, 60 min or above. According to the optimum parameters obtained, Ti was deposited on the inside of the patterned glass cap by magnetron sputtering under a hard mask. Figure 10a shows the integrating of Ti getter in the micro pressure sensor. Here, the vacuum glass cap was formed by a photoresist mask and by wet etching with hydrofluoric acid (HF). The results of the wafer-level packaging of the sensor are illustrated in Figure 10b. The getter was fully activated with the color of bluish-violet (Figure 10c), compared to the initial ash black after it was heated for 6 h at 600 °C. Figure 10d shows an enlarged view of the prepared getter film with a sputtering power 350 W, argon ambient pressure 8 Pa, and sputtering time 10 min at a magnification of 50 K by SEM. The thickness of the Ti getter was approximately 300–500 nm. The results of the preparation and activation indicated that the getter had exerted a suction action to absorb the excess air in the bonding chamber to maintain the high-Q value of the micro resonant pressure sensor.

## 5. Quality Factor Test and Result

**Figure 11:**
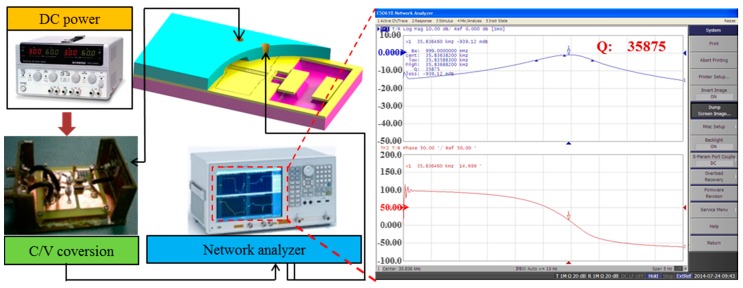
Open loop experimental set-up for the Q test of the resonator. Network analyzer is the main electrical equipment of open loop test.

After the cross-layer anode bonding and the high-quality getter were studied, and the wafer-level package of micro resonant pressure sensor was completed, the Q test system was set up to evaluate the proposed package method in order to verify whether the proposed tri-layer package based on tri-layer anodic bonding and Ti getter could guarantee the high Q value of the sensor and maintain long-term stability. The Q test system is schematically shown in Figure 11, mainly including the C-V conversion circuit, the network analyzer (Agilent E5061B, Santa Clara, CA, USA), and the DC power supply (GPD3303S, Gwinstek, Taiwan). The network analyzer is the core electrical equipment for open loop tests. T/R gain mode was selected in the open loop test, then the segmented sweep frequency was used to find the frequency, and finally the Q of the sensor could be obtained via calculation.

Fifty-seven devices with Q signal were classified by the range of Q. The Q obeyed the normal distribution of N (15,102.33, 8396.094) up to 47,000 and 42 resonators had a Q of more than 10,000, occupying more than 73% of the resonator with the Q signal. Nearly 1/3 of the devices had the Q of more than 20,000, further illustrating the feasibility of the proposed multilayer anodic bonding.

**Figure 12:**
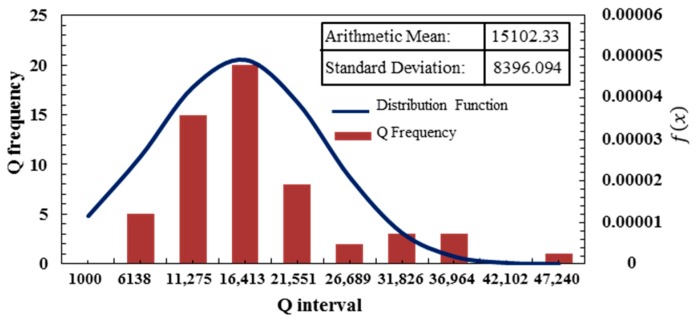
Interval statistics of Q. the number of resonators with Q of more than 10,000 accounts for more than 73% of the total.

**Figure 13:**
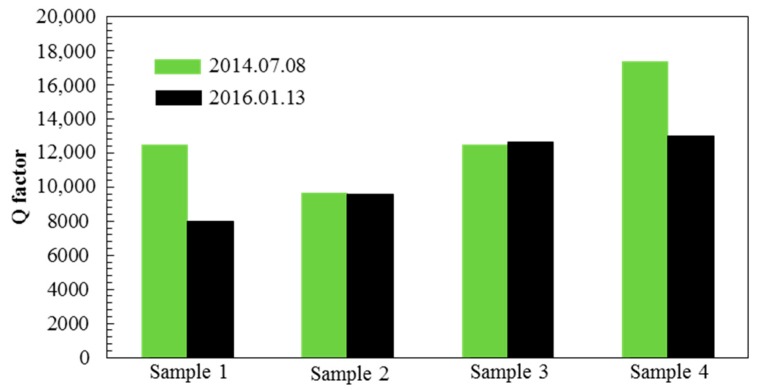
The Q value test of 4 samples with an interval of 1.5 years under the same condition. Sample 1 and sample 4 decreased a little while sample 2 and sample 3 basically unchanged.

With an interval of 1.5 years, four sensitive chips were randomly chosen as samples to measure their Q under the identical condition and the result was showed in Figure 12. The comparative result is showed in Figure 13. The Q of sample 1 and sample 4 decreased, which was interpreted as probably caused by getter deflation, while the other two remained basically unchanged. The result further shows that the proposed level vacuum packaged process is feasible, but still needs to be further optimized in order to improve the yield. This may be because the getter inspiratory capacity reached saturation such a long time after the suction effect. Therefore, it is necessary to have a better getter preparation process to increase the specific surface area in order to enhance its adsorption performance and inspiration time.

## 6. Conclusions

This paper presents one modified tri-layer anodic bonding method to achieve and maintain high-Q of the micro resonant pressure sensor. Due to it being a complicated process and due to the poor bonding quality produced by tri-layer anodic bonding by single-step, the cross-layer anodic bonding technique was adopted to achieve high vacuum package degree by sputtering Al on the combination wafer of the pressure-sensitive layer and the resonance layer. The similar time constant and total charge between the GS bonding by single-step anodic bonding and the GSS bonding by cross-layer anodic bonding indicated that the sputtered Al achieved the ”short circuit” effect as the electrical model of the tri-layer anodic bonding by cross-layer bonding previously mentioned. In addition, the ultimate tensile strength of the tri-layer anodic bonding by cross-layer is 25 MPa and by single-step is lower than 9 MPa, which also showed that the proposed cross-layer anodic bonding ensured better bonding quality. Meanwhile, the Ti getter was also introduced into the vacuum cap layer to maintain the vacuum package degree. The optimal process parameters of sputtering Ti were obtained by measuring the specific surface area and thickness of getter under different process conditions. It was 8 Pa, 350 W, with the thickness of 240 nm and the BET of 50.8695 m^2^·g^−1^. In order to remove the passivation layer of the Ti getter in order to expose its active surface, the optimal activation parameters were also obtained by measuring the oxygen content of the films, which was 600 °C, 60 min or above. The 100% hermetic package showed the reliability and feasibility of the proposed tri-layer anodic bonding. The Q test results showed that the number of resonators with Q of more than 10,000, accounted for more than 73% of the total. Four sensitive chips are randomly selected to test Q under the same conditions with an interval of 1.5 years. Two were slightly lower, while the other two were nearly the same. The value and stability test of Q shows the proposed level vacuum packaged process is feasible and can realize high-Q, long-term stable operation of the sensor.

## Figures and Tables

**Table 1:** The effect of getter film on vacuum in the chamber. Cell A was the sample without getter and Cell B was getter-integrated.

Residual Gas Pressure (mbar)		
	Cell A (Getter-Free)	Cell B (Getter-Integrated)
H_2_	2.76 × 10^−2^	0.00
He	3.71 × 10^−3^	1.63 × 10^−3^
CO	0.00	0.00
N_2_	8.12 × 10^−2^	0.00
CH_4_	4.45 × 10^−3^	2.79 × 10^−2^
H_2_O	0.00	0.00
Ne	1.40 × 10^2^	1.42 × 10^2^
O_2_	8.09 × 10^−1^	0.00
C_2_H_6_	0.00	5.28 × 10^−3^
C_3_H_8_	7.62 × 10^−3^	2.12 × 10^−2^
Ar	7.28 × 10^−3^	0.00
CO_2_	1.33	0.00

**Table 2:** Sputtering experimental parameters used for the first time.

Sample	A	B	C	D
pressure/Pa	0.1	2	0.4	0.4
power/W	200	200	50	350
